# Strangulated right inguinoscrotal hernia with scrotal bowel evisceration

**DOI:** 10.4314/gmj.v59i1.6

**Published:** 2025-03

**Authors:** Mawuena A Kornyoh, Donald T Enti, Vincent Kudoh

**Affiliations:** 1 Surgery Department, Cape Coast Teaching Hospital, Cape Coast, Central Region, Ghana

**Keywords:** hernia, evisceration, bowel evisceration, inguinal hernia, inguinoscrotal hernia

## Abstract

Spontaneous bowel evisceration with umbilical hernia is very rare and has been reported in a patient with chronic liver disease with tense ascites. There has been one reported case of spontaneous evisceration in a patient with inguinal hernia and chronic obstructive pulmonary disease. Here, we present a case of a right inguinoscrotal hernia with spontaneous scrotal bowel evisceration in a patient with no apparent underlying chronic conditions. Management of such a case involves stabilising the patient and avoiding further injury to the bowel, with the definitive treatment being surgical repair. Using a nylon darn repair technique seems better than a prosthetic mesh due to the risk of infection of the latter. The principle of surgical treatment is that the bowel is carefully inspected, a thorough saline wash of the abdomen is done and appropriately repairing the hernia defect.

**Funding:**

None declared

## Introduction

Inguinoscrotal hernias are essentially a common presentation in the surgical department however hernia associated with spontaneous bowel evisceration is a very rare and potentially life-threatening complication.^1^ Common complications of inguinoscrotal hernias include strangulation and incarceration, with spontaneous evisceration being rare. Most spontaneous evisceration of the bowel has been reported in patients with tense ascites, abdominal trauma, and chronic obstructive pulmonary disease and only once in an otherwise healthy patient.^2^ We had not seen a case report of a giant inguinoscrotal hernia with spontaneous bowel evisceration in an otherwise healthy patient until this patient presented to us. Some precipitating factors, such as any event that suddenly increases intra-abdominal pressure, precede the final moment when a rupture occurs; these include lifting heavy objects, coughing, and straining, as seen in constipation.^3^

Even with these factors, there must be a compromise in the scrotal skin layers for the bowel to eviscerate through the scrotal defect. Some possible reasons for this could be the application of strong herbs with the hope of shrinking the swelling^4^, especially with the belief that a scrotal swelling may just be a large carbuncle/furuncle. Another reason could be some form of trauma to the skin like an incision made by non-medical professionals or herbalists.^5^ in an attempt to drain the swelling of fluids, especially because of this belief that it could be a boil.^6^,^7^

The management of eviscerated hernias depends on some factors worth noting such as compromised blood supply and possible contamination of eviscerated bowel were factors that could increase the risk of prosthetic mesh infection in our patient, hence we thought it necessary to use a nylon darn repair method instead of the use of a prosthetic mesh. Nylon darn repair technique is a tension-free type of repair that has been deemed safe, sound, cost-effective and easy for surgeons to perform.^8^,^9^

This case report aims to share with the medical community, a rare and unique case of inguinoscrotal hernia complicated by spontaneous bowel evisceration through the scrotum in an otherwise healthy patient and to describe the uncertainty with the precipitating cause of the scrotal skin breakdown and subsequent bowel evisceration. This case also emphasizes the importance of carefully examining intra-abdominal bowel loops proximal to the hernia sac in the intraoperative period. This report would help to expand the paucity of information regarding spontaneous bowel evisceration as a complication of inguinoscrotal hernias in patients with no known chronic medical conditions.

## Case Report

**Figure 1:**
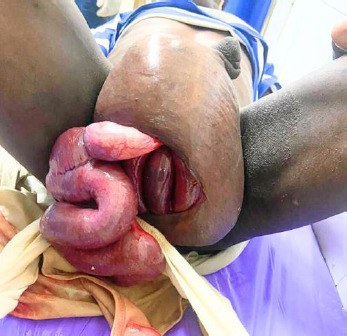
Right inguinoscrotal hernia with bowel evisceration through a break in scrotal layers. The eviscerated bowel appeared healthy

A 54-year-old Ghanaian fisherman living in the Ivory Coast was admitted to the Surgical Department through the Emergency Unit of Cape Coast Teaching Hospital (CCTH) on October 5, 2023. He presented with a scrotal mass with an exposed bowel segment. For over a decade before the presentation, he had had a reducible bilateral inguinoscrotal hernia (with the left more pronounced than the right). He had surgery (at a different facility) for the left one after it became obstructed about a year before the presentation. The right one was not repaired because the hernia defect was small. He had no other known chronic medical conditions. Over the months leading to his presentation, he noticed that the right inguinoscrotal hernia had increased in size and became increasingly difficult to reduce. The morning before the presentation, he felt a wet soft mass in between his thighs which he presumed to be a bowel (Figure 1). Hence, he was rushed to the hospital. There was associated moderate abdominal pain but there was no vomiting.

He reported that his job involved lifting and pulling heavy ropes for fishing as well as lifting heavy buckets of fish. He also reported that he had not sustained any trauma to the scrotum or abdomen; he did not apply any topical application to the scrotum in an attempt to reduce the hernia and had not applied any incision over the scrotal swelling before the evisceration. On assessment, he had rapid shallow breathing (Rate- 36 cycles per minute), was moderately dehydrated and his random blood sugar was elevated (13.6 mmol/L with normal urine ketone and glucose indices), his vital signs were otherwise stable. On examination, he had dry mucous membranes with reduced skin turgor. He received intravenous paracetamol 1 gram 4 hourly, morphine 10 milligrams 4 hourly, and normal saline 1L bolus. A saline-soaked gauze pack was placed over the eviscerated bowel. His urine output was monitored. His blood chemistry showed mild hyponatremia (133.9mmol/L), mild hypokalemia (3.34mmol/L), eGFR 69ml/min/1.73m2, and his haemogram also showed his total white cell count in the upper limit of normal, a normal haemogram. A diagnosis of strangulated right inguinoscrotal hernia with spontaneous bowel evisceration was made. He was quickly prepared for surgery.

During surgery, the eviscerated bowel was normal. A right inguinal approach was made and the content of the hernia was inspected. Some parts of the bowel identified as jejunum, ileum and caecum were gangrenous hence a midline incision was then made where the entire length (165cm) of gangrenous bowel was resected with ileocolic anastomosis done. He had a limited right hemicolectomy with resection and ileocolic anastomosis. A thorough saline wash was done and the inguinal defect was then repaired using nylon darn. The immediate postoperative period was uneventful. He stayed on the ward for 7 days before discharge. During his stay, bowel rest was instituted using Nil Per Os protocol and nasogastric tube feeding, potassium maintenance (60mmol/day), intravenous paracetamol 1 gram 6 hourly, intravenous normal saline 1.5 Litres, Ringer's Lactate 1.5 Litres, and intravenous dextrose normal saline 1 Litre in 24 hours, were administered, monitoring fluid input and output; vital signs remained stable.

His random blood sugar values normalized in the immediate postoperative period. His postoperative haemogram, however, showed moderate normocytic normochromic anaemia, which we believe may have been due to these factors: loss of blood during surgery (estimated blood loss intraoperatively was about 500mL), loss of a significant portion of his bowel, and reduced absorption of nutrients with the presence of diarrhea, among others. He received two units of whole blood to correct this anaemia.

His renal function test revealed normal indices with eGFR being 70ml/min/1.73m^2^. A major complication seen in his postoperative period was diarrhoea usually after meals and before meals hence we restricted his oral intake to 1.5L daily with 1.5L intravenous maintenance fluids ongoing as well as a high protein and high-calorie diet. Oral loperamide 2mg was given due to increasing diarrhoea. He was then discharged on post-op day 7 when his stitches were removed and sent home on oral multivitamins and heamatinics.

At follow-up, three weeks later, he complained of persistent diarrhoea which our team managed. Over 3 months postoperative, his diarrhoea gradually reduced to one bowel movement a day.

## Discussion

A unique phenomenon in this case was that no obvious aetiology underlying the break in all layers of the scrotal skin, permitting the scrotal skin to rupture and for the bowel to eviscerate. We considered the likelihood of a Maydl hernia because the eviscerated bowel appeared healthy. However, during the bowel inspection through the inguinal incision, the bowel proximal to the hernia sac was gangrenous. This type of hernia is usually seen in patients with a long-standing hernia where a significant portion of the bowel drags into the scrotal sac. Surgeons must have a high index of suspicion for a Maydl hernia especially when the hernia is strangulated or obstructed^10^ to avoid the fatal complications associated with leaving behind a gangrenous bowel within the abdominal cavity.

We could not ascertain a direct causative factor for bowel evisceration in this patient, but we can suggest that his long history of heavy lifting as a fish farmer could have contributed.

## Conclusion

Long-standing inguinoscrotal hernias can result in bowel evisceration in patients with no known underlying chronic conditions. Early interventions can help save the eviscerated bowel and reduce morbidity and mortality. It is imperative to seek early health care for inguinoscrotal hernias.
